# "The Hospital" Nursing Mirror

**Published:** 1896-08-15

**Authors:** 


### "The Hospital" Nursing Mirror.

Being the Extra Nursing Supplement of "The Hospital" Newspaper.

[Contributions for this Supplement should be addressed to the Editor, The Hospital, 428, Strand, London, W.C., and should have the word "Nursing" plainly written in left-hand top corner of the envelope.]

### News from the Nursing World.

### ANOTHER JUBILEE.

Many projects are afloat to celebrate the sixtieth year of Queen Victoria's reign. The offering of the women of England ten years ago was most happily expended in the establishment of that most useful institution of the Queen's Jubilee Nurses. It is proposed, if another fund is raised next year, that it should be devoted to the extension of this excellent movement. Such a proceeding would prove doubly welcome to the Queen now that she has become personally acquainted with the admirable work done by the large number of women enrolled under her name.

### CITY OF LONDON UNION INFIRMARY.

The enquiry which is proceeding, as we go to press, in the board-room of the City of London Union Infirmary, is being held by Dr. Downes, Inspector of the Local Government Board. It will deal very fully with the administration of the nursing department at that institution. The employment of counsel by both medical officer and matron have given a serious aspect to the consideration of details of discipline on which there has been friction between the two officers. The incident of the patient being "tied to a bedstead" was satisfactorily shown to have a case, in which a probationer had fastened a broad bandage across a woman's chest "to keep her tidy" till the doctor's visit, as she was so restless she could not remain covered. The probationer explained that she was unaware that she ought to have asked advice before resorting to even the similitude of restraint.

### NURSES' HOME AT WORCESTER.

The scheme for providing a nurses' home in connection with the Worcester Infirmary is slowly progressing. There appears to be still some hesitation in the local mind whether a large sum of money can really be properly spent in the housing of nurses, but fortunately there are others more enlightened at work also who recognise that good service demands proper consideration. We are glad to learn the plans are now to be prepared for this very necessary addition to the Worcester Infirmary.

### DEFINITE AGREEMENTS.

We have often warned nurses tnat in the pursuance of their calling definite agreements are very necessary. It is not always the nurse who is the sufferer through the want of a business compact; but in all cases employed and employer stand in much better relations towards each other when matters are arranged on a business footing. We have recently received a complaint from a parent whose daughter was dismissed from a hospital after signing an agreement to enter into the service of the institution. No one ascertained that the document was a properly executed agreement, and so the nurse had no redress when she found it not to be so. Again, an employer was put to much inconvenience by a nurse who left her case at the end of six days, and contended that she thought this constituted a week. As this matter was brought into court, it was clearly brought home to the nurse that a week was a definitely understood space of time. It is not always desirable to appeal to law when misunderstandings arise, and therefore it is very necessary that engagements of all sorts should be clearly and definitely drawn up.

### NEW NURSES' HOME FOR BRADFORD INFIRMARY.

The Board of Management of the Bradford Infirmary, at their last meeting, on August 7th, decided to forthwith set about the provision of a new home for the nursing staff, to be erected in the infirmary grounds at an estimated cost of from £8,000 to £10,000. After the meeting there was a presentation of medals by the Mayor of Bradford (Alderman Willis Wood) to the nurses who had distinguished themselves in their recent examinations. Mr. Lupton (chairman) afterwards made the welcome announcement of the Board's decision with regard to the new home, and the Mayor spoke in very appreciative terms of "the admirable work performed by the nursing staff of the infirmary," adding that the people of Bradford would be glad indeed to learn that the Board of Management were doing their utmost not only to provide the infirmary nurses with more comfortable quarters, but also to afford them the best facilities for acquiring the most efficient training.

### NURSING ASSOCIATION FOR SUFFOLK.

In the report of a meeting lately held at Ipswich in connection with a County Nursing Association for Suffolk, published in the *Ipswich and East Anglian Times* of the 25th inst., the name of the Rev. A. L. B. Peile, President of the Queen's Jubilee Institute, appears amongst the speakers. We are informed, however, by Mr. Peile that he was not present at the meeting in question.

### A NURSING HOME FOR HASLEMERE.

The proposal to establish district nursing at Haslemere has met with warm support, and a meeting is shortly to be held in favour of it. The local doctors are all ready to support the movement, and the parish council have under consideration a scheme to assist.

### PRIMITIVE PERSONAL HYGIENE.

"Now, Mrs. Williams," said the district nurse "I've made the room clean and your bed looks more comfortable with the fresh quilt and sheets." "Yes, thank'ee, nurse, I feels quite smartened up. What time shall I look for you this evening?" "I am afraid it will be rather late; about seven o'clock, most likely." The busy nurse went off to her next patient, thinking contentedly of the improvement she had made in Mrs. Williams' surroundings. Quite unexpectedly she found herself free at five o'clock. "I

shall be in time to see that she has a comfortable tea," she thought as she hastened back to sick Mrs. Williams. She paused on the threshold in dismay, for a soiled upper sheet and quilt had replaced the snowy-white ones she had so carefully put on a few hours ago, and the clean nightcap had also disappeared. "You've come afore your time, nurse," said the patient, "my daughter was agoin' to get me changed afore you come." "But," protested the puzzled district nurse, "I made you so comfortable this morning and left you looking so nice." "Just so," replied the patient, "I ain't got no fault to find with you adoin' what you fancies you ought to, but you can't expect the likes o' me to waste all them clean things by usin' of 'em when you ain't here to see 'em!"

### NURSING AT CLAPHAM.

There are a certain number of persons who still believe that there are serious grievances of the nurses at the Wandsworth and Clapham Union Infirmary to redress. Our report after the visit of our Commissioner to the infirmary showed that the causes for complaint were of a trivial character. At the last meeting of the Infirmary Committee it was arranged that the combined Relief Committee should inquire into the whole subject with a view to remedy any real grievances that might be discovered, the Local Government Board having stated that there appeared cause for an inquiry. That the accommodation is inadequate for the present staff there can be no doubt, and if the reform is directed remedying this a great improvement will be effected.

### MASTER AND MATRON.

It is really surprising, even in the jurisdiction of red-tapeism, that such obviously impracticable nursing arrangements should still hold good at our workhouses. At Plymouth it has just been ruled that a nurse cannot leave the house without the permission of master and matron, the superintendent of nurses not having the power to grant leave. This state of affairs naturally leads to friction, and elsewhere, in a case where master and matron were not agreed, the nurse applied to the master only, with the result that the matron reported the nurse to the guardians as having disobeyed rules. Here it appeared that the authority of the master was supreme, though the matron in this case was responsible for the nursing. It is obvious with such chaotic regulations that nursing in connection with workhouses is so unsatisfactory as to account for the extreme reluctance of trained nurses to undertake such posts. We do not wonder that the Rev. J. Wyatt expressed doubts at a recent meeting whether the Guardians of Poplar could secure an adequate supply of trained nurses for their infirmary. Nevertheless, we congratulate the Poplar Guardians for advancing in the right direction, and when once it is understood that they are desirous of instituting proper arrangements, their difficulties will steadily decrease.

### WINCHCOMBE COTTAGE HOSPITAL.

A new wing of the Winchcombe Cottage Hospital is to be opened by Mrs. Dent on Thursday, September 10th. On the same day a sale of work will be held at the Hospital, for which articles will be very gladly received by Miss E. McCaul, hon. secretary and matron.

### NIGHT NURSING IN THE WORKHOUSE.

It did not need the special commission of the *British Medical Journal* to teach the initiated that much remains to be done before the sick poor are uniformly well cared for in the union infirmaries of our country. Nevertheless, we have heard fewer advocates for State-supported hospitals from the lay public since the revelations brought to light by that commission have been published. But much as there is yet to be done in respect to English unions, the state of affairs in Ireland is still worse. Only this week we read in a Dublin paper of a union infirmary containing sixty beds, the nursing staff for which consists of one nurse (untrained), assisted by five female paupers and two male paupers, both infirm. The women helps have to scrub ten wards and do the laundry work of the infirmary in addition to the usual ward work. The chief male help is aged seventy-four, and his hearing is defective. The nurse in charge locks up at ten o'clock, and does not visit the patients again until seven in the morning unless summoned. Thus patients who are seriously ill are left to the care of infirm and incapable paupers during the whole night. We are glad to hear that in this case the Local Government Board has taken the matter up. The present nurse has a salary of £16 a year.

### NURSES AT TOTNES.

Matters at Totnes Union Infirmary are still in a most unsettled condition. It appears probable that the Workhouse Nursing Association will have to sever their connection with the infirmary, as the conditions required by them do not seem likely to be fulfilled by the Guardians. The proposal that the nurses should not have the duty of receiving vagrants imposed upon them has been rejected, and this improper proceeding still holds good.

### INTERFERING RELATIVES.

We regret to find that we referred in mistake to the Lewisham Union Infirmary instead of to the Lambeth Union Infirmary in writing under this heading last week.

### SHORT ITEMS.

Under the auspices of the North Riding Nursing Association, a garden *fête* was held on Monday in Kiplin Park by kind permission of the Hon. Admiral and Mrs. Carpenter.—A measure which will be doubtless much appreciated is being taken to provide a nurse for the female patients attending the Exeter Dispensary.—A *fête* and bazaar were held at Poltimore Park last Wednesday and Thursday, under the patronage of Lord and Lady Poltimore, on behalf of the county fund for the training of district nurses.—Steps are being taken to provide a properly qualified person as parish nurse for Fisherton, Salisbury.—The entertainment given by Harrod's employés in aid of Guy's Hospital on Saturday, August 8th, was a great success.—Through the kindness of Miss Jenkins, the resident matron, a reception was held recently at the Eye Hospital, Bristol.—The Tsar of Russia is an active, and the Tsarina an honorary, member of the Russian Red Cross Society.—Dr. Robinson, Medical Officer at the Mile-End Infirmary, has been granted a gratuity of £25 by the Guardians, in recognition of his successful instruction of the nurse probationers.

### Hygiene: For Nurses.

By John Glaister, M.D., F.F.P.S.G., D.P.H.Camb., Professor of Forensic Medicine and Public Health, St. Mungo's College, Glasgow, &c.

### XIX.—EXERCISE—REST—RELAXATION—IN RELATION TO MAINTENANCE OF HEALTH.

The animal body is a machine built and fitted for the generation, storage, and expenditure of energy. Like other machines of a complicated kind, the various parts do not only perform their work in their own special way, but they have a correlation with one another, and with the entire machine. Linking together the mind and the body is the nervous system, by which the will may send from the brain to muscle messages with lightning speed. When, for instance, it is desired to bend the elbow, a message is sent to the biceps muscle by means of the nerves which cause this muscle to contract, and the necessary action is produced. During contraction the muscles become harder and thicker, whereas during relaxation, they become softer. In this simple movement there is expenditure of energy, because work has been done. Regular use of muscles produces an increase in their bulk and strength. For this reason, the arm of the blacksmith becomes "brawny," and his muscles "strong as iron bands." This condition of a muscle—when increased in size proportionally to the general muscular standard of the body—is called hypertrophy (Greek, *hyper*—over, and *trophè*—nutrition). During the process of enlargement of the muscle the supply of nutriment is greater than the waste, hence there is growth; and when the maximum growth has been attained this supply must be equal to the waste, hence there is maintenance of condition. On the other hand, if the expenditure of energy be continuously greater than the muscle can bear another condition may come on, called atrophy (Greek, *a*—under, *trophè*—nutrition). Here the waste is greater than the supply of nutriment. Complete inaction of a muscle induces the same state, as in a limb which has been "up" in splints for a length of time.

From these facts, then, we may gather two principles for our guidance, viz., first, that over-exercise is harmful, and, second, that under-exercise is equally baneful, and, as a natural corollary, follows the additional fact, that adequate exercise is necessary for the maintenance of bodily health. It is approximately true that in health the excretory products are equal to the waste of the body, due to the expenditure of energy, and to the excess of food taken. If an amount of food be taken which enables a healthy man to sustain hard labour over a period of time, the supply will probably just equal the demand; but if an enforced period of inactivity follow with the same supply of nutriment, the supply will become greater than the demand, the elimination of effête products less complete, and, sooner or later, discomfort or illness will follow. Exercise increases the functional activity of every organ of the body. The number of respirations per minute is increased, consequently the amounts of $CO_2$, watery vapour, and organic matter from the lungs are also increased; and, in like manner, the functions of the skin, kidneys, liver, and stomach become more active. This usually leads to development of appetite, by the amount of which the food supply required by the body is, or ought to be, regulated.

The practical aspects of exercise and work are of great importance. From the physiological point of view it matters little whether the energy expended be in pleasure or in business. The two main evils of to-day are over-training on the one hand, and insufficient exercise on the other. Successful training ought to consist in the attainment of maximum muscular development compatible with health. This is not the same for all men, but is relative to the individual. We cannot all be Samsons; at the same time, the man of small physique may be stronger muscularly than he of larger bulk. Over-training involves grave risks to health. It involves undue strain on the heart and large blood-vessels; hence athletes of all classes, whether of the field, of the ring, or of the river, are most liable to break down from diseases of these organs. But under-exercise is probably the more prevailing evil. Temptations are all in this direction. The railway-train, the omnibus, the tram-car, and penny steamers have all been invented to save time, which, to-day, in the race for wealth, has a money value. It is difficult to convince the eager business man that walking to business will pay him better in the end than any of the aforementioned modes of progression. The leisurely business man in the end accomplishes more than his colleague who rushes through his business day at high pressure. The increasing nervous diseases, the flow of insanity, the sudden deaths from heart diseases, and many crippled lives from other causes are the costs of this unceasing restlessness.

Exercise for the young is all-important. Out-of-door games of all kinds, suitable to the physique and temperament, ought to be encouraged. Usually children adapt themselves to the kind of exercise most suited for them. Sex is not now the barrier it formerly was in the prevention of girls and women taking part in athletic exercises—tennis, cricket, croquet, golf, fishing, cycling, and even shooting, now form the pastime of both sexes. Walking is the only general form of exercise which threatens to become a lost art in our city populations; and yet there is no more healthful form of exercise. A walking tour, to a person in training, amid picturesque scenery, is one of the most invigorating of holidays. A day on the river, rod in hand, is a stimulant of the most bracing character; and the like is true of the round of golf on the upland links, or the game on the bowling green. Persons who work in confined positions, those of sedentary occupation, can maintain good health by walking, by moderate gymnastic exercises, or by any of the aforementioned modes. All work and no play makes Jack not only a dull, but also an unhealthy boy. The healthy mind in the healthy body can only be attained and maintained by consideration being paid to the needs of both brain and muscle respectively, and in ratio to the relative calls upon each.

Rest.—After labour comes rest. Exertion brings with it the sense of fatigue, which is nature's signal for rest, and for the need of nutrition. Every machine has a natural term of life, and this is as true of a locomotive as of a man. The tear and wear, which eventually cannot be repaired, renders the machine useless. In a man, expenditure of energy of brain, or body, or both, calls for rest. The close interassociation of brain and body means that fatigue of the one involves, to a certain extent, fatigue of the other. In unskilled labour there is greater expenditure of muscular than mental energy; in skilled, there is expenditure of both. For the mental worker the energy expended is principally nervous, and little or none of it muscular.

Rest, therefore, for these classes of workers, is but a relative term. The manual worker needs bodily rest; the mental, brain rest. In the former, moderate mental action, as in reading, would be beneficial, and in the latter, moderate muscular action is required. Change of mental labour is a form of rest, for there is a certain sense of relief experienced in changing the field of study. But this is only temporary; for, after all, while the current of thought is changed, it is but switching the current into a new channel, and the thought-generating machine is working all the while. Rest of any organ depends largely upon its function, both as to amount and kind. In walking we do not hop upon both legs at once, as does the kangaroo, and in the alternate use of the limbs nutrition and alteration of energy-expenditure take place. Even the heart, though constantly in action, has its periods of rest in the momentary pauses between each beat, and special provision has been made for rest of the brain in sleep.

Rest is the animal expression of relief from the sense of fatigue. The tired animal, like the tired man, lies down to rest its weary limbs.

Relaxation, however, is only possible to the thinking animal, man. During relaxation there is a certain amount of expenditure of mental or bodily energy, or both, but it partakes of a form which is only calculated to divert the mind or titillate the senses. We look at pictures, we converse, listen to lectures or music, or attend the theatre, from the sense of pleasure these things produce. Rest and relaxation, therefore, are inseparably linked together when one dissociates himself from the business of his life, but they may not be present in equal degree at the same time. The object of both, however, is recreation. The fitful daily periods of rest and relaxation from daily toil doubtless serve a useful purpose, but they are unsuited entirely to sustain the healthful body over long periods of continuous work. Holidays ought to form part of the scheme of life of every man, and they ought to be for him holy-days, set apart as religiously for the work of physical recreation as his Sabbaths are for spiritual recreation. Especially is this true for the denizens of populous places, but equally necessary for all. A holiday conduces to better health and longer life, and if it be devoted to the regeneration of the mind and body in the contemplation of nature or art, the works of God or those of man, accompanied the while with agreeable physical exercise, it will achieve this purpose. "Hobbies" and "fads," of whatever kind, are most healthful and helpful. They exercise a beneficial reactive influence in taking the individual out of the rut of routine, and they make the wheels of life go more smoothly. Whatever its form, be it carpentering, model making, photography, book or stamp collecting, autograph hunting, chemistry, or any other kindred science, such as botany or geology, let the hobby be pursued leisurely, profitably, and consecutively, and let it contrast as much as possible with the serious business of life. For the mental worker, let it be an occupation of the hands, which will cultivate skill, and which can best be carried out in the open air. His object ought to be to allow the brain to lie fallow for a season, and to reasonably develop his bodily physique against the time he returns to his pen and his books.

### Ethics of Nursing.

(*Continued from page* clxv.).

### The Duty of the Nurse to Her School.

Sec. 1.—It is the duty of the nurse to be loyal to the school from which she graduated. From the fact that the nurse has remained long enough in the school to accept its certificate, she has tacitly admitted that she owes it her allegiance, and should avoid adverse criticism on its management. A nurse can best do honour to her school by her personal conduct, and by the high character of her professional work.

Sec. 2.—She should always wear a complete uniform of the school when on duty either in the hospital or when in charge of a private case.

Sec. 3.—Every member of the Alumnæ Association should feel it a duty to further the interests of the society not only by attendance at the regular meetings and by the payment of fees, but also by giving her hearty support to all work proposed by it, and by interesting the public in such work in all legitimate ways.

### The Duty of Nurses to Each Other.

Sec. 1.—A nurse should avoid adverse criticism of another nurse, especially to doctors or patients, unless she knows the nurse to be unreliable, and is called upon for an expression of opinion when the question of sending such a nurse to a patient is involved.

Sec. 2.—The unity and dignity of the nursing profession demand that members of one school cultivate a courteous recognition of all other schools in good standing, of their work and of their graduates.

### District Nursing in Holland.

The following letter of a Dutch District Nurse trained by the Q.V.J.I., which was recently published in "Nursing Notes," will interest all nurses :—

"It is a great pleasure to me to give you a little account of my experiences in district nursing in Holland. I started work on October 15th, 1894, in Fevolle, entirely on the principles of the Queen's Institute. A local committee, consisting of four gentlemen and two ladies, was formed, which meets once a month and watches the work with the greatest interest.

"I made my first appearance in a little back street, where some children were suffering from catarrhal pneumonia, after measles. People not accustomed to be nursed in their own homes were quite surprised at first. It was not easy to get them to put on clean bed-clothes, or to get them washed and the rooms ventilated. Many people objected to have a stranger in the house, some thought it an insult, because they were so dirty (you did not expect this in Holland, I suppose), but gradually the poor began to appreciate the work, they did not feel ashamed any longer; and after half a year, I daresay, the work had taken a pretty good hold on the people.

"The work was also very much appreciated by the doctors. They often told me how great a value it was to them in their practice. They were often quite surprised at the change in the rooms. Fevolle is divided into four districts, and is a nice, kindly looking place, having 33,000 inhabitants; some parts are very quaint and old. At first one does not get an impression of much poverty, but all the same there are a good many poor people hidden in little slums and back streets. Generally the poor are living in small cottages, consisting of one room, where the dinner is cooked, the washing done, and the whole family has to sleep. Consequently a good deal of phthisis is going on. Very often the windows do not open at all, and ventilation is kept up by the door, people going in and out, which is not the very best thing for a sick person, of course. I often tried to keep all the room fresh and sweet by hanging up a sheet and leaving the door open, if the street was not too noisy. The floors are of red stone, sprinkled with white sand. In each room are one and two, and sometimes three box beds for the people to sleep in; these box beds are often very damp and stuffy, so I soon bought a bedstead, which I used as soon as there was room to put it down, and many a bad case has been nursed in it. During the first year 174 applications were made, 160 cases nursed, and 1,199 visits were paid.

"As Amsterdam wanted to start district nursing on the same principles, a lady came to Fevolle for a short training, and since October 15th, 1895, she is working in Amsterdam, where the work has been received also very favourably, 661 visits being paid during four months. In the meantime Rotterdam was preparing for district nursing, and since February 17th I am trying to get the work planted there. If the work gets on, we hope to get a training home in Rotterdam for district nurses on the principles of Q.V.J.I. Now we are expecting our first pupil here."

### Nurses in 1896—Their Quarters, Hours, and Food.

### ST. THOMAS'S HOSPITAL.

### I.—Terms of Training.

It is a little difficult to get a clear grasp of the somewhat peculiar circumstances which surround all the nursing arrangements at St. Thomas's Hospital, carried on as they are entirely through the Nightingale Training School, the matron of the hospital holding also the post of superintendent of the Nightingale School, but the management of the two institutions being kept entirely distinct and separate the one from the other.

The regulations as to training at St. Thomas's Hospital, under the Nightingale Fund, were originally drawn up for two separate and distinct classes of women, paid probationers, who were not ladies, and who, after their training, became staff nurses, and special, or paying probationers, who were gentlewomen (that being an essential qualification), and from whom the sisters, or heads of wards, were (and are still) always chosen. This system naturally excluded many most suitable women from receiving training at St. Thomas's Hospital, on account of inability to pay for their maintenance during the first year, for which period a payment of £30 is required, and service for two years after leaving the school. Ladies are now allowed to enter the Nightingale Home as ordinary probationers, but it is with the understanding that they are not eligible for promotion to the post of ward sister at St. Thomas's though they may be placed in that position in some other institution. It is a peculiarity of the Nightingale agreement that its nurses are bound to its service for four years, but during that time they may be placed by the committee in any institution, other than St. Thomas's Hospital, where the matron is a Nightingale nurse. According to the printed rules of the Nightingale School, it still stands that "at the close of a year, their training will usually be considered complete," in the case of both classes of probationers; this is really misleading, as their training is not practically considered to be complete under four years, for which term they are, in the case of the paid probationers, bound by agreement; actually, the "one year" refers to their residence in the Nightingale Home. The special probationers are not promoted to the position of ward sister until they have had experience in day and night staff nurse's work.

### II.—Hours of Work and Times off Duty.

Probationers' hours on duty are as follows: Wards 7 a.m., dinner 12.45 to 1.30, off duty 10.30 to 12.45, or 3 to 5, tea 5 to 6 p.m., then wards till 8.30, when they return to the home, having supper at 9 p.m., bed at 10. Actual working hours are therefore nine and three quarters. Probationers, on two days in the week, are also released from the wards for one and a half or two hours for class instruction, or lecture attendance. Once a month they have a whole day off duty, not entering the wards at all, but they are not permitted to sleep away from the home without special leave. The staff nurses have ten hours on duty daily, beginning work at 7 and leaving their wards at 9.30 p.m., with two hours for recreation, one hour for luncheon, and half-an-hour each for dinner, tea, and supper. Nurses are off duty on Sundays first and third week from 3 to 10 p.m., and every Sunday they are off duty from 9 a.m. to 12.30 p.m., being expected to attend chapel during part of that time. Two hours and a-half extra leave is granted on one afternoon in the week, and once a month leave of absence from 6 p.m. to 10 p.m. the following day, being allowed to sleep away if desired. Three weeks holiday is given in summer and one week in winter. Sisters have nine hours forty minutes on duty daily, from 8 a.m. to 9.45 p.m. They have two hours off duty daily, with twenty minutes for luncheon, 45 for dinner, and thirty for tea and supper. In addition to this they are off duty on alternate Sundays from 4.30 p.m., (once a month from 10 to 1, to allow opportunities for church-going outside the hospital) from 1.30 to 6 p.m. one afternoon in the week, and leave of absence once a month from 6 p.m. to 10 p.m. the following day. Annual holidays are four weeks in summer and two in winter. Night nurses are on duty from 9.30 p.m. to 8.30 a.m., with time off for two meals during the night. They have three hours for recreation every day, and once in two months two nights off duty. At St. Thomas's it is customary for a year's night duty to be taken at a time, instead of alternate three months. In Miss Gordon's opinion it tries the nurses less to get thoroughly accustomed to the routine, and settle down to a reversal of ordinary hours in this way rather than having to re-adjust their way of living four times in a year.

### III.—Meals.

The nurses breakfast at 6 40 a.m., dinner is served at 12 o'clock, the matron or her assistants presiding, and supper is at 8.30 p.m. Luncheon in the morning, and afternoon tea are taken in the ward kitchens. The first-year probationers return to the Nightingale Home for tea. The dietary consists of, for breakfast, tea or coffee, bacon, eggs (which are ordered especially from the country), and cold meats. Luncheon is of milk and bread and butter. For dinner and supper meat and vegetables, puddings, cheese, and in the summer fresh fruit are provided. Night nurses have a meat meal before going on duty, taking with them eggs or sardines, with bread and butter, milk, and tea and coffee for night meals. Their dinner is at 9.30 a.m., the same as that provided for the day staff. Nurses off duty at tea time can make their own tea in their sitting-rooms, a gas stove for boiling the water being placed on each dormitory floor. The food for these and the night meals is kept in special cupboards in the ward kitchens. The sisters have breakfast, luncheon, and tea served in their own rooms by the wardmaid. Dinner and supper are laid in the dining room. Miss Gordon dines alternately with the sisters and staff nurses, whose meals are at different hours.

### IV.—Salaries and Uniform

Probationers in their first year receive during their year of training payment in money and clothing to the value of £14 on the following footing, thus: Clothing, costing about £4; payment at the end of the first quarter, £2; at the end of the second quarter, £2 10s.; at the end of the third quarter, £2 10s.; at the end of the fourth quarter, £3. During their second year the nurses receive from £20 to £22 per annum, with a gratuity of £2 per annum from the Nightingale Fund during the first three years, and for the fourth year and after £26 per annum. Two shillings a week is allowed to all the staff for washing. Sisters' salaries vary from £32 to £50 in the ordinary wards. The sister of No. 8 Block receives £60 to £80 per annum. The Nightingale Fund gives £4 per annum to the sisters, in recognition of their work in training the probationers. None of the staff are required to wear outdoor uniform. Indoor uniform, consisting of four washing dresses, with eight aprons, four caps, three collars, two waist belts, and a shawl for wearing through the rather cold corridors, is provided.

### V.—Nurses' Quarters.

The accommodation for the nursing staff is divided. The Nightingale Home is for the probationers in their first year. Here they have separate bed-rooms, and a charming dining-hall and sitting-room, pleasantly and comfortably furnished. There is also a class-room, and a sick-room. The home is a separate block, entered from the long, main colonnade on one side, and from the Lambeth Palace Road on the other. At the end of their first year's training the probationers take up their abode in the hospital itself, where on the top floor of each pavilion are separate rooms, originally cubicles, for the nurses working in its wards. These little bed-rooms are small, but fairly comfortable. On this floor, in each block, is also a small sitting-room, and a bath-room and lavatory. There is on the ground-floor of the hospital, close to the matron's office, a large general sitting-room, provided with papers and magazines, and here is the dining-room for the use of sisters and nurses, the Nightingale probationers returning to the home for meals.

### A Book and its Story.

"IN SCARLET AND GREY."

The daughter of so distinguished a man of letters as the late Lord Houghton, Mrs. Henniker has had an intellectual environment of no mean order, but she has fairly distinguished her own personality through the literary qualities of her present volume. That she knows her world can be said of the authoress of "In Scarlet and Grey*" as it cannot be said of all writers of fiction in the present day; knows it, not in any restricted sense, but in a wide all-embracing manner, through the medium of a mind ever ready to observe life in its tragic and in its humorous aspect. For the most part the stories which comprise the volume under discussion are grave in tone, serious-minded, and pathetic, but throughout her pages Mrs. Henniker's ready humour is never absent. This quality in her writings prevents her even approaching to any morbidness, born of a one-sided view of life. Her tragedy is all the more tragedy because of her full understanding of what comedy is, and her stories, collected under their "Scarlet and Grey" title, compel our interest, even if when we do not criticize their characteristics.

"The Spectre of the Real," written conjointly with Mr. Thomas Hardy, and "In the Infirmary," are perhaps the two most uncommon in the collection, but the strongest amongst Mrs. Henniker's tales is called "At the Sign of the Startled Fawn."

"The Startled Fawn" is an English Wayside Inn, and here the story, a local tragic incident, is recounted by the landlady to a casual passer-by, the substance of which he gives us himself.

"It was the week after Ascot, twenty years ago, and a day of warmth, sunlight, and colour. A group of more or less heated men were sitting outside the porch discussing the past races, the coming harvest, and the doings of the neighbourhood in general. The social qualities and conduct of the greatest land proprietor in the district, Lord Eddington, were freely commented upon, in a strain that was hardly flattering.

"The language of the said great man being described as capable of 'lifting the tiles right off yer roof,' and what with his short neck, and that vulgar face of his under his white hat, that makes it look ever so much redder—why, any one'd be astonished to see 'im take a first-class ticket to go racing." From discussing my lord the speakers fell to discussing my lady, with whom they expressed much sympathy.

"Ah! Miss Edith Brunner never did a sillier thing than when she went to church with that foul-mouthed young brute, lord or no lord," remarked one of the speakers. "Young Tom Drax is wild after her still, I'm told."

As they were thus conversing the landlady was hastily summoned to the back door, where a young woman was asking for her. The young woman, wearing a gown of tumbled print and a straw hat, was standing half propped up against the kitchen table. She was white—livid almost.

"You're faint," said the landlady kindly, seeing that the girl breathed hard, and that the perspiration stood on her forehead in large drops.

"Yes—faint—and very, very ill," murmured the stranger. "I've come such a long way in the broiling sun." She spoke in quick gasps. The landlady noticed that her skirts were torn, and that pieces of briar, and here and there stems of long grass adhered to her petticoat. "Can you let me rest here?" said the girl. She bowed her head over the wooden table, and tears fell from her eyes.

"Poor soul, you're in sad trouble!" the compassionate landlady exclaimed, and there and then offered her all the practical help which suggested itself to her mind.

Then the girl raised her head and said with great earnestness, "Will you send a note for me? You must swear to me, though, that no one else shall know where or to whom it has gone." She drew out a sealed envelope as she spoke.

"If I were not ill I'd go on now," she whispered; "but I should only faint by the roadside. You have a kind face. I throw myself on your pity. I am married, and am flying from my husband. I put on one of the maid's dresses and I came quickly away on foot; no one saw me but one of his grooms, I might have——. I swear I'll never go back to him, for nothing in this world or the next!"

"Look here!" she tore open her dress. Across her white neck stretched a bruise, turning purple; a still larger mark was on her wrist. "Those are some of the places where he's struck me," said the stranger piteously. "Now will you help me? O God! you must and surely will."

The tears stood in the landlady's eyes; "I'll do all you ask, ma'am."

The girl handed her the note; it was addressed to, "T. Drax, Esq.'

Then the landlady had an idea, and she foresaw great trouble. She spoke in great perplexity to the wretched wife. "Send it, or I'll kill myself before to-morrow!" exclaimed the latter, and she won her cause.

Then Edith Eddington was taken up the back staircase. In the spare bedroom she flung herself upon the little sofa, overlooking the corn fields, and a distant silvery glimmer of the river.

Two hours later, a man drove up in a dog-cart to The Startled Fawn, a very tall, very handsome young fellow. Just a few snatches of tremulous conversation, scattered heart-broken utterances, reached the landlady through the door. She heard the man swear an eternal protection to the woman, and then another man's voice, loud and course in tone, said, "So Drax is here." He pushed past the group, arrived at the top of the stairway; he laughed aloud, a harsh, metallic laugh, cracking his hunting whip as he went.

The men closed on one another in fierce encounter, then the people below heard a shriek as Edith ran across the room to separate the two. The three had unconsciously drawn near the head of the staircase in their movements, and when Lord Eddington hurled her backwards she lost her balance. With one loud cry of fear and anguish she fell down all the flight of oak steps, striking her head sharply against the iron-bound chest that stood upon the landing. Then she lay still, a narrow red stream trickling slowly from her forehead over the polished boards.

And Tom Drax kept his word. After a while, the woman he loved recovered, though her mind had gone, but he took her to his people's home and guarded her tenderly. The finish of the tragedy should be read in Mrs. Henniker's own words, so charmingly and pathetically is it told.

* "In Scarlet and Grey." Mrs. Henniker. London: John Lane, 1896.

### Everybody's Opinion.

[Correspondence on all subjects is invited, but we cannot in any way be responsible for the opinions expressed by our correspondents. No communications can be entertained if the name and address of the correspondent is not given, or unless one side of the paper only be written on.]

### NURSING IN THE COLONIES.

Mrs. Piggote, hon. secretary Colonial Nursing Association, writes: I shall be grateful if you will mention in your widely-read paper that I find it quite impossible to answer until the autumn the innumerable letters I have received from persons, nurses, and others who wish to go abroad. The Crown Colonies that require nurses have not yet had time to communicate with us. Those nurses who enclosed a stamped and directed envelope will receive answers in October; this will give us time to hear from some of the colonies we have communicated with. Will you kindly draw the attention of nurses to the fact that we supply private nurses only, and do not attempt to find posts abroad for matrons, superintendents, male and female asylum attendants, probationers who wish to train in colonial hospitals, &c. Will correspondents who have written to me about such posts take this as an answer. Suitable applications from nurses will be entered in our books, and full particulars and forms of application will be sent to them as we hear of vacant posts.

### NURSES' HOURS.

"A Lover of Sunshine" writes: Upon reading the article *re* the Middlesex nurses in your last issue of The Hospital, I was surprised that greater stress was not laid upon the fact of the long hours of duty required of the ordinary probationer in the Middlesex Hospital. Surely it is more than can possibly be expected that any woman possessed of the average amount of bodily strength could under such circumstances perform her duties wich any degree of intelligence and interest! The time allowed by late pass would appear to be more profitably spent in bed than elsewhere. What a luxury that one day during the first year must be to the weary, overtired probationer. It is to be hoped that those interested in hospitals and their management will do their best in the near future towards shortening the hours on duty for the Middlesex nurses. May I be allowed to suggest that, through your valuable medium, The Hospital, it would be interesting to have some correspondence from matrons and superintendents *re* the advantages and disadvantages of "days off" duty to probationers?

### Minor Appointments.

Lewisham Union Infirmary.—Miss Hetty Dixon has been appointed Assistant Matron at this institution. She was trained at King's College Hospital, where she held the appointment of staff nurse. She has since been ward sister at the Lewisham Infirmary.

Salisbury Infirmary.—Nurse Agnes Mary Lancaster has been appointed head nurse of the male surgical ward at Salisbury Infirmary. She received her training at this institution, and has since held for a year the position of staff nurse at the Coventry and Warwickshire Hospital.

Brook Fever Hospital, Shooters' Hill.—Mrs. Mary Ada Lockitt has been appointed assistant matron at this new hospital. Mrs. Lockitt received her training at the Nightingale School, St. Thomas's Hospital, where she afterwards held the posts of staff and charge nurse. Since then she has acted as night superintendent of nurses at the Gore Farm Fever Hospital, Dartford. We congratulate Mrs. Lockitt on her appointment.

### Notes and Queries.

The contents of the Editor's Letter-box have now reached such unwieldy proportions that it has become necessary to establish a hard and fast rule regarding Answers to Correspondents. In future, all questions requiring replies will continue to be answered in this column without any fee. If an answer is required by letter, a fee of half-a-crown must be enclosed with the note containing the enquiry. We are always pleased to help our numerous correspondents to the fullest extent, and we can trust them to sympathise in the overwhelming amount of writing which makes the new rules a necessity. Every communication must be accompanied by the writer's name and address, otherwise it will receive no attention.

### Queries.

(139) *A Nurse s' Club.*—Please tell me if there is not in London a club or home where nurses could stay for three or four nights at less expense and more conveniently than at an hotel?—*Policy Nos.* 39 *and* 4,604.

(140) *Army or Navy Nursing Service.*—Please tell me what steps I should take to obtain appointment in the Army or Navy Nursing Service.—*M. P. Ireland.*

(141) *Skin Discoloration.*—Some months since I had the misfortune to fall face downwards upon a cinder path, making a nasty jagged cut on the bridge of my nose, long since perfectly healed, but there remains a blue discoloration in or under the skin. I should be obliged by any suggestions as to the best way of restoring the skin to its original colour.—*H. H.*

(142) *General Training.*—Please tell me what would be the best hospital in London to go to for one year's general training, where probationers are paid.—*Subscriber.*

(143) *Psychological Examinations.*—Where can I get information concerning the psychological examinations for asylum nurses?—*A. B.*

(144) *Midwifery Nursing.*—How can I set about obtaining midwifery work? I hold the L.O.S. diploma.—*Gladys.*

(145) *Holidays.*—Please give me the address of comfortable lodgings in Newquay. I believe this has been given in a former number of The Hospital.—*Kathleen.*

### Answers.

(139) *A Nurses' Club* (*Policy Nos.* 39 *and* 4,604).—Apply to the Nurses' Hostel (Miss C. J. Wood), 27, Percy Street, Tottenham Court Road; Miss Ely, 25, Norfolk Terrace, Hyde Park, W.; or Miss Culverhouse, 18, Royal Avenue, Chelsea, S.W.

(140) *Army or Navy Nursing Service* (*M. P. Ireland*).—Write direct to the Director-General, Army Medical Department, Victoria Street, Westminster, and the Director-General, Naval Medical Department, Avenue House, Northumberland Avenue, S.W.

(141) *Skin Discoloration* (*H. H.*).—Your question is one for a medical man. Why do you not consult your doctor?

(142) *General Training* (*Subscriber*).—You will not obtain "one year's training" in any good hospital and receive a salary. You will find full particulars of the terms of training at London and provincial hospitals in "How to Become a Nurse," Scientific Press, 428, Strand, W.C. Why do you not enter a good training school for the usual two or three years? You will not regret the time thus spent if you really mean to do good nursing work. *Full name should accompany all queries.*

(143) *Psychological Examinations* (*A. B.*).—The address of the Medico-Psychological Association is 11, Chandos Street, London, W. Write to the secretary for all particulars.

(144) *Midwifery Nursing* (*Gladys*).—Do you belong to the Trained Nurses' Club and Midwives' Institute, 12, Buckingham Street, Strand, W.C. Write to the secretary, enclosing stamped envelope for reply.

(145) *Holidays* (*Kathleen*).—Write to Mrs. North, Erncliffe, Newquay. The address is given in this column for July 25th.

### Appointments.

### MATRONS.

Hospital for Infectious Diseases, Cardiff.—Miss Margaret Lawrence Hay has been appointed Matron at this hospital. Miss Hay is a Nightingale nurse, trained at St. Thon as's Hospital, where she has also held the appointment of staff nurse for 1½ years. For one year she was senior sister at the Cardiff Sanatorium. Miss Hay has our best wishes for success in her new work.

West Malling Isolation Hospital.—Miss Mary M. Fisher has been appointed Nurse Matron of this hospital. She was trained at the Royal Hants County Hospital, Winchester, where she also undertook matron's duties. She has since held the appointments of charge nurse at the Royal Sheffield Hospital; head nurse at the Sanitary Hospital, Bournemouth; matron of Halstead Cottage Hospital, Essex; matron of the Nurses' Home, 38, Nottingham Place, London, and assistant matron of the Hanover Institute for Nurses, London. Miss Fisher's testimonials are excellent.

### Death in Our Ranks.

We regret to announce the death of Miss Annie L. Bird, M.R.B.N.A., from diphtheria, contracted in the faithful performance of duty. Miss Bird was for five years the much esteemed and highly valued matron of the Royal Hospital for Sick Children, Edinburgh, a post which she resigned at the end of 1894. She was much beloved, and her loss will be deeply felt by her many friends.

### The Book World for Women and Nurses.

[We invite Correspondence, Criticism, Enquiries, and Notes on Books likely to interest Women and Nurses. Address, Editor, The Hospital (Nurses' Book World), 428, Strand, W.C.]

Bradshaw's Dictionary of Climatic Health Resorts, Hydropathic Establishments, &c. (London: Kegan Paul, Trench, Trübner, and Co. Price 3s. 6d.)

We have received a new and improved edition of this useful dictionary, which cannot fail to be extremely helpful to the many seekers after health at mineral watering-places, both at home and abroad. The introductory chapters contain sound advice, both on the choice of a health resort and the way to get there. The dictionary portion is full and complete, and in the cases of the more frequented places, such as Davos-Platz, Schwalbach, &c., minute details are given for the benefit of intending visitors. The names of doctors and hotels are supplied, and have been made fully up-to-date.

Girlhood and Wifehood. By A Surgeon Accoucheur. (London: The *Family Doctor* Publishing Company, Catherine Street, Strand. Price 2s. 6d.)

We fail to agree with the author of "Girlhood and Wifehood" that his book is indispensable to girls and mothers. There is nothing in it of use which has not been placed before the public already in other handbooks, and there is much which might have been omitted with advantage. In matters of advice we do not always agree with the writer. The physiological part of the book is far too sketchy to be of any real use, and in our judgment the book will do very little in fulfilling the author's object, viz., that of doing something towards "improving the stamina" of his race.

### MINOR PUBLICATIONS.

### A New Birthday Book.

Marcus Ward and Co. have just brought out a bright little volume in the form of a new birthday register, with selections from humorous writers. "Merry Thoughts," which is the name it will go by, will, we prophecy, attain a wide popularity, for it is not only a very daintily got up little volume but it is full of entertaining quotations from the writings of wits and humorists, chosen with consummate skill. On each page there are spaces for sixteen names to be inscribed, with four clearly-printed verses or sentences of a genuinely amusing order on the opposite side of the page. It is good to laugh, as Mr. W. M. Praed reminds us in the introductory poem to "Merry Thoughts."

"Oh! trust me, whatsoe'er they say,
There's no thing half so good as laughing;
Never sigh when you can sing,
But laugh like me at everything."

### Skertchly's Physical Geography.

This popular little volume has just been revised up to date by John H. Howell, B.A., and now, in its twenty-eighth edition, is offered to the public at the price of 1s. Few text-books have attained a wider popularity than has this particular one, and now that it is revised it is even more worthy of its popularity. Whilst the general plan of the manual has been retained, its scope is considerably increased; and the astronomical parts have been entirely re-written, consistently with a wide development of present-day education. "Skertchly's Physical Geography" is illustrated by maps and diagrams, and is a wonderful volume at the price. It is published by Murty, of Ludgate Circus Buildings, E.C., under the personal supervision of which firm the well-known "Science and Art Department" series of text-books has been brought out.

"The London Manual for 1896-7."—The "London Manual" has now passed into a second edition, and we are not surprised to hear that this volume, which is a complete municipal year-book, has found universal favour. In a limited space an amazing amount of information is contained concerning public bodies in the metropolis and the suburbs, the editor's intention with regard to his work being to adapt it to serve as a handy reference book, "not only to the large class who are associated with public work as members and officials, but also to citizens in their relation to public authorities." It seems to those of us who have it as a ready reference book now, that we are surprised we have ever got on without the "London Manual." It is published for one shilling at the offices of "London," the leading municipal journal, 125, Fleet Street, E.C.

"Everybody's Bible Dictionary."—To judge a dictionary conscientiously it would be almost, if not quite, as arduous a task as compiling it, but it seems to us that "Everybody's Bible Dictionary," published by Saxon and Co., 23, Bouverie Street, E.C., supplies a want long felt. This volume is offered to the public at a price placing it within the power of all, and, whilst prepared with consummate reference and care, is in a handy serviceable compass. The printing is excellent, and Mr. Walter Baches, the editor, is to be congratulated on the excellence of his work.

"Cassell's Natural History."—Messrs. Cassell are inexhaustible in their educational endeavours, and now they include "Natural History" among the list of their histories which are appearing in weekly and monthly parts. Part I. of their new venture is before us for review. This edition is to be completed in twenty-six weekly parts. The present number contains about 2,000 illustrations, with descriptive and authoritative letterpress. It is a wonderful sixpenny-worth, and is beautifully got up, no expense or trouble being spared in the "history" itself, or in the reproduction of the plates, which are excellent. A series of new full-page plates is being expressly prepared for the edition, reproduced from photographs of representative animals in the Zoological Gardens, taken during the present month.

